# A Novel Case of Deep Temporal Artery (DTA) Embolization for Recurrent Subacute-Chronic Subdural Hematoma

**DOI:** 10.7759/cureus.38164

**Published:** 2023-04-26

**Authors:** Ashish Kulhari, Farah Fourcand, Amrinder Singh, Haralabos Zacharatos, Siddhart Mehta, Jawad F Kirmani

**Affiliations:** 1 Neurology, University of Missouri Kansas City School of Medicine, Kansas City, USA; 2 Medicine, Kansas City University of Medicine and Biosciences, Kansas City, USA; 3 Neurology, Research Medical Center, Kansas City, USA; 4 Neurology, Hackensack Meridian John F. Kennedy (JFK) University Medical Center, Edison, USA; 5 Neurology, United Health Services (UHS) Binghamton General Hospital, Johnson City, USA; 6 Neurology, Hackensack Meridian John F. Kennedy (JFK) Medical Center, Edison, USA

**Keywords:** particle embolization, endovascular embolisation, middle meningeal artery embolization, deep temporal artery embolization, chronic subdural hematoma (csdh)

## Abstract

Subdural hemorrhage (SDH) is a common neurological disease. In past, SDHs were managed either conservatively (non-surgically) or with surgical evacuation (burr hole versus craniotomy) depending on the severity. Surgical evacuation has major challenges including high recurrence rate, stoppage and reversal of antiplatelet or anticoagulation agents, risk of general anesthesia and surgery in elderly patients with multiple comorbidities. Given the above challenges, embolization of the distal branches of the middle meningeal artery (MMA) has recently emerged as an excellent alternate to surgical evacuation or conservative management. To the best of our knowledge, there is no literature on the embolization of the deep temporal artery (DTA) for subacute-chronic SDH. We report the first case of recurrent subdural hematoma post MMA embolization that was successfully treated with embolization of DTA.

## Introduction

Subdural hemorrhage (SDH) is a common neurological disease. Incidence of new SDH is estimated to be around 60,000/year by 2030 [1]. Pathophysiology of acute and chronic SDH is very different. While most acute SDHs are venous in origin, chronic SDHs are arterial in etiology [2]. No standard guidelines exist currently for the management of chronic SDH. Small SDHs (<10 mm in thickness with <5 mm midline shift) are generally managed conservatively (non-surgically) while larger SDHs undergo surgical evacuation with burr hole or craniotomy [1]. Major challenges with surgical evacuation include high recurrence rate, stoppage and reversal of antiplatelet or anticoagulant agents that increase risk of thromboembolic complications, risk of general anesthesia and surgery in elderly patients with multiple comorbidities [1].

Given the above challenges, embolization of the distal branches of the middle meningeal artery (MMA) has emerged as an excellent alternate to surgical evacuation or conservative management. First case of MMA embolization for chronic SDH was reported by Mandai et al. in 2000 [3]. Since then multiple case reports and case series have strongly supported the safety and efficacy of MMA embolization for chronic SDH [4-14].

There is no case in literature on the embolization of the deep temporal artery (DTA) for subacute-chronic SDH. We report the first case of subdural hematoma recurrence post MMA embolization that was successfully treated with embolization of DTA. We also describe in detail our technique of MMA and DTA embolization.

## Case presentation

**Figure 1:**
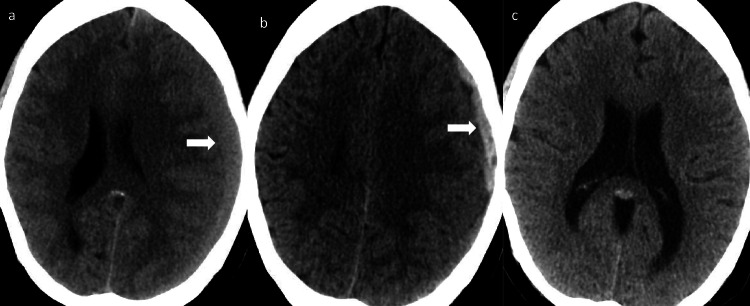
a: CT head on admission showed 14 mm left frontal-parietal subacute subdural hemorrhage with 9 mm left to right midline shift. b: One-month post middle meningeal artery embolization CT head showed 10 mm acute left frontal subdural hemorrhage. c: Six months post deep temporal artery embolization CT head showed completely resolved subdural hemorrhage.

**Figure 2:**
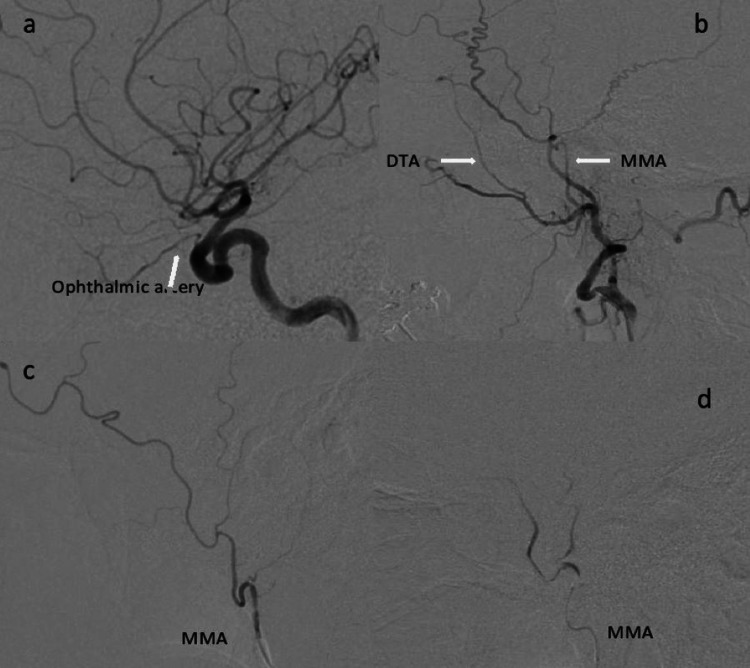
a: Left ICA angiogram (lateral view) shows robust ophthalmic artery. b: Left ECA angiogram (lateral view) shows the MMA and well-developed DTA. c: Left MMA angiogram (lateral view) shows frontoparietal and temporal branches. d: Post MMA embolization angiogram (lateral view) shows lack of flow in the distal branches. ICA, internal carotid artery; ECA, external carotid artery; MMA, middle meningeal artery; DTA, deep temporal artery

**Figure 3:**
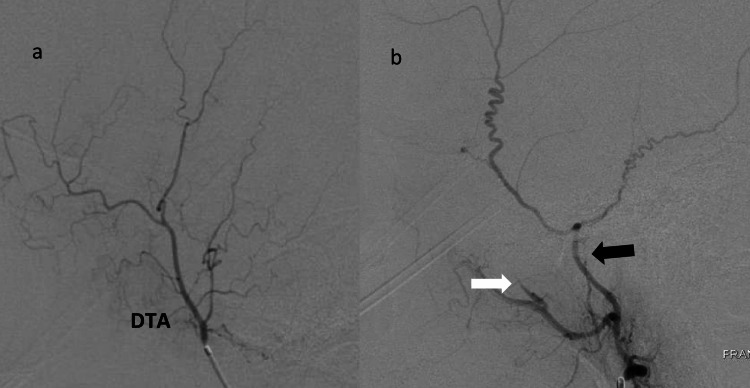
a: Left deep temporal artery angiogram (lateral view) shows distal branches. b: Post embolization angiogram (lateral view) shows stasis of contrast in the proximal deep temporal artery (white arrow) and proximal middle meningeal artery (black arrow).

A 57-year-old woman with past medical history of breast cancer status post mastectomy, left ulnar vein thrombosis (diagnosed about four months prior to admission, on rivaroxaban), hypertension, hypothyroidism, was admitted to our hospital for progressively worsening headaches after a motor vehicle accident two weeks prior to admission. CT head revealed 14 mm left frontal-parietal subacute SDH with 9 mm left to right midline shift (Figure 1a). Anticoagulation was held on admission. She underwent successful embolization of left MMA with 100-300 microns Embosphere® Microspheres (Merit Medical, South Jordan, UT) with complete obliteration of the frontal and parietal branches of MMA (Figure 2). While being off the anticoagulation, the patient developed acute right calf deep vein thrombosis (DVT) causing pulmonary embolism. Anticoagulation was therefore resumed. The patient was discharged home on full anticoagulation. One-month post embolization CT head revealed 10 mm acute on chronic left frontal SDH (Figure 1b). Anticoagulation was stopped and an inferior vena cava (IVC) filter was placed. Because of the history of acute DVT and pulmonary embolism off the anticoagulation, the patient was scheduled for urgent digital subtraction angiography (DSA) with an intent to embolize left DTA as it was noted to be well developed during previous embolization and was thought to be feeding the subdural membrane. She underwent successful embolization of left DTA with 100-300 microns Embosphere® Microspheres (Figure 3). Post embolization, therapeutic anticoagulation was resumed. One-month post embolization CT head showed resolving SDH. Six months post embolization CT head showed complete resolution of the SDH (Figure 1c). The patient made excellent recovery and remained headache free at one year post embolization follow up.

Embolization technique

Both the embolization procedures were performed under conscious sedation (midazolam and fentanyl) and local anesthesia. Right common femoral artery was accessed with a 5 French 23 cm Cordis sheath (Cordis, Miami Lakes, FL). After sheath placement, 30-50 units/kg of intravenous heparin was administered. A 5 French MPD Envoy (Codman Neurovascular, Raynham, MA) guide catheter was used to select the internal carotid artery (ICA) ipsilateral to the SDH. Intracranial biplane angiography of ICA was performed (Figure 2a). Close attention is paid to size and flow in the ophthalmic artery. External carotid artery (ECA) was then selected and biplane angiography was performed (Figure 2b).

Under road map guidance, Prowler Plus microcatheter (Codman Neurovascular, Raynham, MA) was used to catheterize the MMA and positioned proximal to the bifurcation of frontoparietal and squamous/temporal arterial branches. Biplane angiography was then performed to confirm the position of microcatheter and to rule out any dangerous anastomosis (Figure 2c). Similar to MMA embolization technique, microcatheter was positioned at the proximal DTA and biplane angiography was performed to rule out any dangerous anastomosis (Figure 3a). At this point, a homogenous mixture of particle suspension is achieved using equal parts (1:1) of contrast (Visipaque 320, Iodixanol, GE Healthcare, Marlborough, MA) and packaged 100-300 microns Embosphere® Microspheres (Merit Medical, South Jordan, UT).

After ruling out dangerous anastomosis, 100-300 microns particles were administered in a slow pulsatile manner to embolize MMA and DTA. We believe that smaller particles provide better distal penetration in the subdural membrane resulting in better angiographic embolization outcomes. Particles were administered until stasis of anterograde flow and/or reflux around the microcatheter tip was noted. Microcatheter was then cleaned with normal saline. Post embolization MMA/DTA angiography demonstrated lack of flow into the distal branches (Figures 2d,3b). 

## Discussion

Subdural hemorrhage is a common neurological disease. With high prevalence of antiplatelet and anticoagulation agents, increasing traumatic brain injury and aging population incidence of new SDH is estimated to be around 60,000/year by 2030 making it the most common disease requiring neurosurgical intervention [1]. Pathophysiology of acute and chronic SDH is very different. While most acute SDH are venous in origin, chronic SDH is arterial in etiology [2]. Chronic SDH develops due to constant leakage of blood products from the fragile neo-vasculature of the subdural capsule. Subdural capsule forms overtime from the inflammatory cells and fibroblasts that migrate from dura due to chronic inflammation from broken down blood products [1-2]. Histological and angiographic evidence have confirmed distal branches of MMA to be the main arterial source of the subdural capsule [2].

No standard guidelines exist currently for the management of chronic SDH. Asymptomatic or patients with minor symptoms, with chronic SDH measuring less than 10 mm in greatest thickness with less than 5 mm midline shift, are generally managed conservatively. Patients with severe symptoms or larger chronic SDH, undergo surgical evacuation with burr hole or craniotomy [1]. There are some major challenges with surgical evacuation including high recurrence rate (2%-37% based on various observational studies), stoppage and reversal of antiplatelet or anticoagulant agents, risk of general anesthesia and surgery in elderly patients with multiple comorbidities [1]. Holding antiplatelet or anticoagulant agents for conservative management and reversing antiplatelet/anticoagulants for surgical evacuation puts these patients at high risk for thromboembolic complications depending on the underlying pathology. 

Given the above challenges, embolization of the distal branches of the MMA has emerged as an excellent alternate to surgical evacuation or conservative management. First case of MMA embolization for chronic SDH was reported by Mandai et al. in 2000 [3]. Since then there have been multiple case reports and case series strongly supporting the safety and efficacy of MMA embolization for chronic SDH [4-14]. Studies quoted have used various embolization agents including polyvinyl alcohol particles (150-250 μm), N-butyl-2-cyanoacrylate (NBCA), gelatin sponge, coils, and Onyx for embolization of MMA for chronic SDH [3-13]. Recently Okuma et al. used 300-500 microns Embosphere® Microspheres for MMA embolization [14]. 

There is no case in literature on the embolization of the DTA for subacute-chronic SDH. To the best of our knowledge, this is the first case report on embolization of DTA for subdural hematoma. In our article we describe a unique case of subacute SDH who underwent successful MMA embolization first but required embolization of the DTA a month later because of recurrence of the SDH.

Given the acute SDH post MMA embolization was localized anteriorly, we think the reason for failure of MMA embolization in our patient was because predominant blood supply to the anterior part of subdural membrane was coming from the prominent DTA. This brings us to an important question whether all prominent DTA should be embolized along with the MMA for chronic SDH. Currently there is lack of data to support this assertion. More case reports/series to corroborate our findings are essential.

Our patient made excellent neurological and radiographic recovery and continued to do well almost a year after her embolization procedures. In describing this case, we hope to make neurointerventionalists aware of possible significance of prominent DTA in subacute-chronic SDH.

## Conclusions

Middle meningeal artery embolization has emerged as an excellent alternate to surgical treatment for chronic SDH. There is no literature on feasibility, safety, and efficacy of DTA embolization for SDH. We report the first case of recurrent subdural hematoma post MMA embolization that was successfully treated with embolization of DTA.
